# Expression Status of UBE2Q2 in Colorectal Primary Tumors and Cell Lines

**Published:** 2014-03

**Authors:** Sayed Mohammad Shafiee, Atefeh Seghatoleslam, Mohsen Nikseresht, Seyed Vahid Hosseini, Mahvash Alizadeh-Naeeni, Akbar Safaei, Ali Akbar Owji

**Affiliations:** 1 Department of Biochemistry-Recombinant Protein Laboratory, School of Medicine, Shiraz University of Medical Sciences, Shiraz, Iran;; 2 Cellular and Molecular Research Center, Yasuj University of Medical Sciences, Yasuj, Iran;; 3 Colorectal Research Center, Shahid Faghihi Hospital, Nemazee Hospital, Shiraz University of Medical Sciences, Shiraz, Iran;; 4 Department of Internal Medicine, School of Medicine, Shiraz University of Medical Sciences, Shiraz, Iran;; 5 Department of Pathology, School of Medicine, Shiraz University of Medical Sciences, Shiraz, Iran;; 6 Endocrine and Metabolism Research Center, Nemazee Hospital, Shiraz University of Medical Sciences, Shiraz, Iran

**Keywords:** UBE2Q2, Colorectal Cancer, Cell Line, Gene Expression

## Abstract

**Background: **Activation of the ubiquitin-proteasome pathway in various malignancies, including colorectal cancer, is established. This pathway mediates the degradation of damaged proteins and regulates growth and stress response. The novel human gene, UBE2Q2, with a putative ubiquitin-conjugating enzyme activity, is reported to be overexpressed in some malignancies. We sought to investigate the expression levels of the UBE2Q2 gene in colorectal cell lines as well as in cancerous and normal tissues from patients with colorectal cancer.

**Methods:** Levels of UBE2Q2 mRNA in cell lines were assessed by Real-Time PCR. Western blotting was employed to investigate the levels of the UBE2Q2 protein in 8 colorectal cell lines and 43 colorectal tumor samples.

**Results:** Expression of UBE2Q2 was observed at the level of both mRNA and protein in colorectal cell lines, HT29/219, LS180, SW742, Caco2, HTC116, SW48, SW480, and SW1116. Increased levels of UBE2Q2 immunoreactivity was observed in the 65.11% (28 out of 43) of the colorectal carcinoma tissues when compared with their corresponding normal tissues. Difference between the mean intensities of UBE2Q2 bands from cancerous and normal tissues was statistically significant at P<0.001 (paired* t* test).

**Conclusion: **We showed the expression pattern of the novel human gene, UBE2Q2, in 8 colorectal cell lines. Overexpression of UBE2Q2 in the majority of the colorectal carcinoma samples denotes that it may have implications for the pathogenesis of colorectal cancer.

## Introduction

Colorectal cancer (CRC) is the third most common type of non-skin cancer worldwide and the third cause of cancer-related death in the Western world.^1^^,^^2^ The molecular defects in CRC can be due to changes that result in increased activity of oncogenes or to changes that lead to loss of function of the tumor-suppressor genes.^3^ A large number of colorectal tumors show mutations in the KRAS oncogene as well as in the APC, p53, and SMAD4/DPC4 tumor-suppressor genes. An increasing number of mutated genes have been identified in CRC.^3^^,^^4^ These genes encode proteins with essential roles in CRC carcinogenesis.^4^ These proteins are shown to be regulated by the ubiquitin-proteasome system (UPS). This system regulates various cellular processes by targeting proteins for activation, degradation, or localization at specific intracellular sites.^4^^,^^5^ Ubiquitin-mediated degradation of proteins is comprised of steps mediated by ubiquitin-activating enzymes (E1s), ubiquitin-conjugating enzymes (E2s), and ubiquitin ligases (E3s). The E1 enzyme activates ubiquitin and then transfers it to E2s. Subsequent conjugation of ubiquitin with the specific protein substrate is catalyzed by E2s alone or by E2s together with E3s. Aberrations in the UPS lead to a variety of pathological outcomes and hence are targets for therapeutic intervention in cancer and some other pathologic conditions.^6^^-^^8^ Research has begun to identify the contribution of E2s to tumorigenesis. Various E2 proteins have shown to be closely linked to the cell cycle progression and, hence, tumorigenesis. Two of the E2 enzymes with a role in cancer are UbcH10 (also known as UBE2C or UBC4) and UBE2S (also called E2-EPF), both of which work with E3 ligase APC/C (Anaphase Promoting Complex/Cyclosome) in the regulation of the cell cycle.^9^ UbcH10 plays a role in cell cycle progression and checkpoint control.^10^ This protein is known to be required for APC-dependent ubiquitination of mitotic cyclins.^11^^-^^13^ Other E2s with a role in cancer are reviewed elsewhere.^14^ UBE2Q2 is a novel human gene that belongs to the UBC2 family of enzymes. Isoforms of this gene exist in a variety of species. UBE2Q2 was also designated as UBCi (i for implantation), because its expression changed in the epithelial cells at implantation sites in the rabbit endometrium.^15^ Eenzymatic assays for ubiquitin thioester construction in vitro have shown that UBE2Q2 has a functional role as a ubiquitin-conjugating enzyme.^16^ UBE2Q2 gene is located on chromosome 15 (15q23) and has 13 exons distributed over 57.6 kb. Its cDNA is 2939 bp long and has an open reading frame (ORF) of 1339 bp, which codes for a protein of 375 amino acids.^17^^,^^18^ UBE2Q2 has shown to covalently bind ubiquitin in vitro. In vivo inhibition of this protein is also shown to result in early mitotic arrest and cytotoxicity in cells treated with microtubule-inhibiting agents.^18^^,^^19^

Given the importance of the UPS in cell cycle control, we assessed the expression of UBE2Q2 in a cohort of CRC specimens and cell lines.

## Material and Methods

*Cell Culture*

Colorectal cell lines HT29/219, LS180, SW48, SW480, SW742, SW1116, HCT116, and Caco2 (National Cell Bank of Iran Pasture Institute, Iran) were cultured in a humid incubator at 37°C, under an atmosphere of 5% CO2 and in 10% fetal calf serum (Cinagen, Iran) containing media (Biosera, UK) of either RPMI 1640 (HT29/219, SW480, SW742, SW1116 and HCT116) or DMEM (LS180, SW48 and Caco2). 

*RNA Extraction and Quantitative Real-Time Polymerase Chain Reaction*

Confluent monolayers of the colorectal cell lines, each in 25-cm^ 2^ (T-25) cell culture flasks, were treated with two ml of TriPure Isolation Reagent (Roche Applied Sciences, Switzerland). Total RNA was then extracted according to the manufacture’s protocol. RNA quantity and quality were assessed by ultraviolet spectrophotometry. The integrity of RNA was confirmed using agarose gel electrophoresis. Reverse transcription was performed with 1 μg of RNase-free DNase-treated total RNA and random primer using RevertAid First Strand cDNA Synthesis Kit (Fermentas, USA). Quantitative real-time polymerase chain reaction (qRT-PCR) was done using a 7500 Real-Time PCR System (Applied Biosystems, USA) with SYBR Green^®^ PCR Master Mix (Applied Biosystems, USA) according to the manufacturer’s instructions. The PCR reaction mixture contained 3 μl of cDNA (approximately 150 ng), 1 μl of 5 μM solutions of each of the forward and reverse primers, and 12.5 μl of SYBR Green^®^ PCR Master Mix in a total volume of 25 μl. Primers were designed by using the Primer3 software (http://frodo.wi.mit.edu/primer3/) in order to amplify the exon-exon junction containing regions according to our previous study.^20^ The specificity of the primers was verified by Blast analysis at NCBI, and by analysis of agarose gel (2.0% w/v) electrophorogram as well as melting curves (Tm) of the amplified products. The housekeeping gene, RPLP0, was used to normalize for RNA loading.

The primer sequences were 5’-CCGTGG GTAGTGGTTGATCT-3’ and 5’-AGC GATTCC GCATCGTCAGT-3’ for UBE2Q2 gene and 5′-GAAGGCTGTGGTGCTGATGG-3′ and 5′-CCGGATATGAGGCAGCAGTT-3′ for RPLP0. Thermocycling conditions for SYBR Green consisted of a denaturation step for 5 min at 95ºC, followed by 40 cycles of 95ºC for 2 sec and 60ºC for 30 sec. For all the runs of both genes, a set of 10-fold serial dilutions of the cDNA was used to generate a standard curve. All qRT-PCR assays were linear within this concentration range, with correlation coefficients (r^ 2 ^)>0.999. The data were analyzed by using the standard curve method.^21^ Amounts of UBE2Q2 mRNA were normalized to the levels of RPLP0 mRNA for each sample.

*Tissue Sample Collection and the Clinicopathologic Data of the Patients*

Human CRC tissues were obtained from Shahid Faghihi Hospital (Shiraz, Iran). Forty-three colorectal tumor samples and the corresponding histologically normal tissues were obtained by needle biopsy (12 cases) or derived from surgical resections (31 cases). The patients underwent biopsy sampling or surgical procedures, respectively, for the diagnosis or treatment of cancer. They did not recieve any medication or radiotherapy before surgery or biopsy sampling. The samples were from the advancing edges of the tumors excluding the necrotic centers. Normal samples were collected from the farthest margin of the surgical resections or biopsy samples. The tissues were stained with hematoxylin/eosin and were reviewed by well-experienced pathologists in parallel. All the tissues were frozen in liquid nitrogen within approximately one hour of excision and were stored at -70°C until performing the assay.

*Tissue and Cultured Cell Protein Extraction and Western Blotting*

The tissue samples (normal and cancerous) were lysed in extraction buffer containing 150 mM sodium chloride 1.0% NP-40 (V/V), 50 mM Tris, pH 8.0, and protease inhibitor cocktail (Roche, Germany). The lysates were sonicated at 0°C for 30 sec and then maintained in constant agitation for 2 h at 4°C and finally cleared by centrifugation. To extract protein from the cultured cells, the same protocol was used with 30-min constant agitation at 4°C. Protein concentration was measured using Bradford reagent. Electrophoresis (Mini-PROTEAN Tetra Cells BioRad, USA) of 30 μg protein samples was performed on 12.5% discontinuous polyacrylamide gel, and the proteins were transferred onto nitrocellulose membrane (PROTRAN Nitrocellulose transfer Membrane Whatman) at 25 V for 18 h in cold room (Mini Trans-Blot Cell BioRad USA). The membranes were blocked for 1 h in 5% dried fat-free milk, at room temperature, and incubated with specific primary antibodies diluted (1:2,000) in blocking solution at 4°C overnight. UBE2Q2 polyclonal rabbit antiserum was generated against the peptide LPTGQNGTTEEVTSEEC corresponding to amino acid sequences 125-140 of UBE2Q2.^20^ The blots were washed three times in PBS-Tween (PBS-T) and incubated with specific secondary antibodies coupled to HRP (HRP-conjugated goat anti-rabbit IgG, Abcam, USA) (1:2,500) at a concentration of 1 µg/ml in 2% (W/V) BSA in PBS-T. All the samples were also blotted for β-actin (1:1000) to normalize the amounts of protein. A chemiluminescent substrate (Chemiluminescent Kit BioRad, USA) was used for detecting the bands on the membranes. Light emission was captured by exposing the membrane to X-ray films.

Relative expression levels of the UBE2Q2 protein in the colorectal tissue samples were reported as the ratio of the levels of the UBE2Q2 protein in the cancerous tissues to those in their normal counterparts. The level of the UBE2Q2 protein was assessed by densitometric quantitation of the intensity of the signal from the UBE2Q2 band in relation to that from the actin band using Gel-Pro Analyzer software (version 6.0, Media Cybernetics, Silver Spring,. MD, USA). The intensity of the signal from the actin band was used as internal control.

*Statistical Analysis*

The densitometric data of the UBE2Q2 protein expression in the cancerous tissues and their normal counterparts were analysed using the paired *t* test (SPSS 13 statistical package software). The data were considered significant at P<0.001.

## Results

*Expression of UBE2Q2 mRNA and Protein in Colorectal Cell Lines*

**Figure 1:**
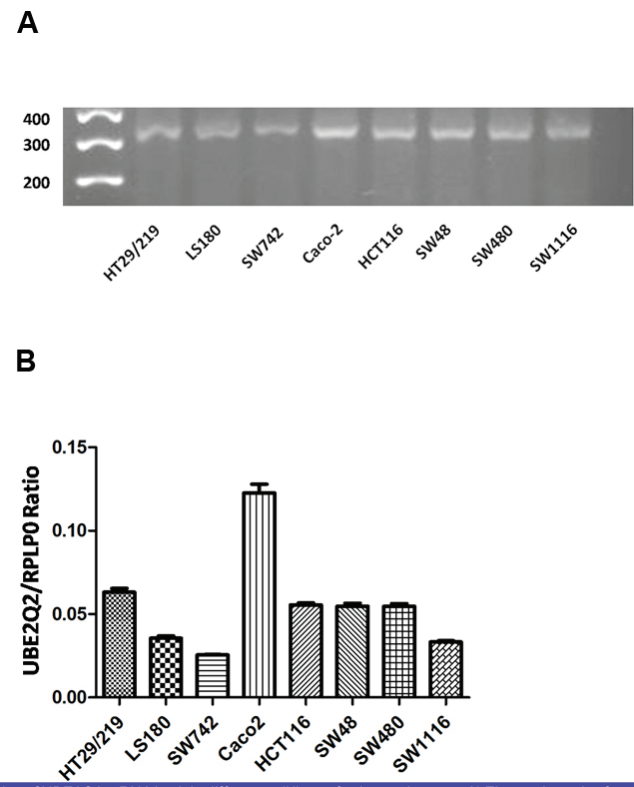
Expression of UBE2Q2 mRNA in eight different cell lines of colorectal cancer. A) Electrophoresis of reverse transcriptase-polymerase chain reaction products ( 317 bp products). B) Quantification of mRNA expression by quantitative reverse transcriptase- polymerase chain reaction. Data are the ratio of the expression levels of UBE2Q2 mRNA to those of RPLP0.

**Figure 2:**
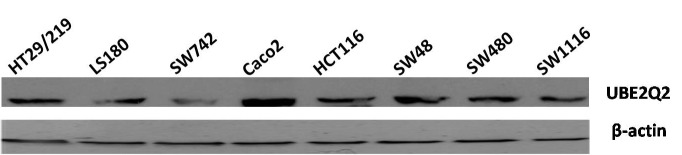
Expression pattern of UBE2Q2 protein in the different colorectal cell lines. UBE2Q2 antibody was used in immunoblot assays against extracts from eight colorectal cancer cell lines. This antibody detected ~43 KDa protein bands.

Colorectal cell lines HT29/219, LS180, SW742, Caco2, HTC116, SW48, SW480, and SW1116 were shown by real-time PCR to have expressed UBE2Q2 mRNA (figure 1A). The levels of UBE2Q2 mRNA relative to those of RPLP0 were determined in these cell lines by using q real-time PCR. As is shown in Figure 1B, cell lines SW742 and Caco2 expressed the least (0.0255±0.00) and highest (0.1224±0.00) levels of UBE2Q2 mRNA, respectively. The expression of UBE2Q2 at the protein level was also assessed by western blot analysis using an anti-UBE2Q2 rabbit antisera generated previously in our laboratory.^20^ As is shown in figure 2, cell lines SW742 and Caco2, respectively, showed the least and the highest levels of UBE2Q2 immunoreactivity.

*Expression of UBE2Q2 Protein in Colorectal Tissues*

**Figure 3:**
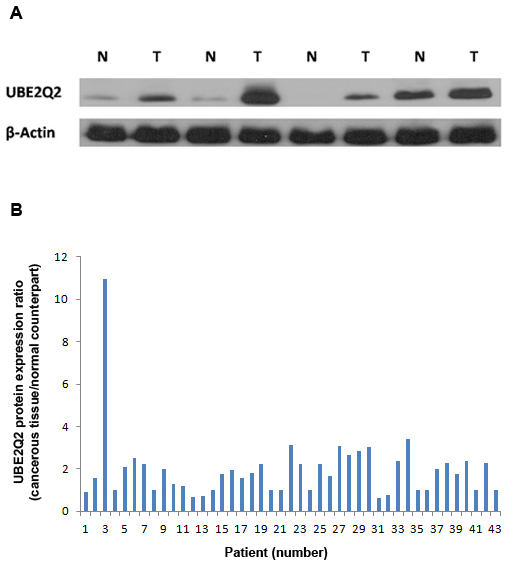
Expression of UBE2Q2 in colorectial tumors and their corresponding normal tissues. A) Immunoblot analysis using UBE2Q2 antibody (detecting ~43 KDa protein bands) against the extracts from four normal human colorectal tissue samples (N) and their corresponding cancerous counterparts (T). Anti β-actin antibody was used to normalize differences in the amount of protein that was loaded into wells. B) Relative expression levels of the UBE2Q2 protein in the colorectal tumors from 43 patients. Data are presented as the ratio of the levels of UBE2Q2 protein in the cancerous tissue to those in its normal counterpart. The level of the UBE2Q2 protein was assessed by densitometric quantitation of the intensity of the signal from the UBE2Q2 blot in relation to that of the actin band as control.

Tissues from the cancerous part of 43 colorectal tumors along with their normal counterparts were subjected to western blot analysis for the UBE2Q2 protein. The individual characteristics of the colorectal cancers are summarized in table 1. Increased levels of UBE2Q2 immunoreactivity were observed in the cancerous tissues related to 28 (65.11%) of the specimens when compared with their corresponding normal tissues (figure 3). No significant changes were observed in the cancerous cells of 10 (23.26%) of the specimens as compared with their corresponding normal tissues (figure 3). Five (11.63%) of the samples showed lower expression levels of UBE2Q2 in their affected cells. UBE2Q2 bands in western blots from 43 cancerous and their corresponding normal tissues revealed intensities of respectively 4957±591 and 3305±451 (mean±SEM) arbitrary units. These differences were statistically significant at P<0.001 (paired *t *test).

**Table 1:** Clinicopathologic information of the 43 patients with colorectal cancer

**Variables**	**Frequency (%) (n=43)**
Gender	
Male	23 (53.5)
Female	20 (46.5)
Age groups	
<60 years	22 (51)
≥60 years	21 (49)
Tumor location	
Colon	15 (34.9)
Rectum	15 (34.9)
Rectosigmoid	5 (11.6)
Sigmoid	8 (18.6)
Tumor differentiation	
A) Adenocarcinoma	40 (93)
Well differentiated	29 (67.4)
Moderately differentiated	5 (11.6)
Poorly differentiated	3 (7)
No comment	3 (7)
B) Mucinous adenocarcinoma	3 (7)
Tumor configuration	
Circumfrential and fungative	1 (2.3)
Fungative	2 (4.65)
Infiltrative	5 (11.6)
Polypoid	3 (7)
Ulcerative	6 (14)
Ulcerative and circumfrential	1 (2.3)
Ulcerative and infiltrative	2 (4.65)
No comment	23 (53.5)
Lymph node invasion	
Negative	22 (51.1)
Positive	6 (14)
No comment	15( 34.9)

## Discussion

Carcinogenesis in CRC is a multistep event that includes a progressive accumulation of genetic alterations in multiple genes whose protein products are regulated by the UPS.^3^^,^^4^ Here, we report the expression pattern of a novel E2-conjugating enzyme, UBE2Q2, in colorectal carcinoma cell lines and colorectal primary tumors. Our results showed that all the colorectal carcinoma cell lines tested express UBE2Q2 protein and also its transcript. Under our experimental conditions, the line Caco2 showed the highest levels of both UBE2Q2 mRNA and protein, whereas SW742, LS180, and SW1116 showed relatively lower levels of expression. This difference may be due to the variation in the characteristics of these cell lines. There are properties in each cell line which are used to classify CRC cell lines such as morphological markers, gland formation, and modal chromosome number.^22^^,^^23^ Thus, SW1116, which expresses low levels of UBE2Q2, falls into Group III of the classification made by Drewinko et al.,^22^ while a high UBE2Q2-expressing cell, like SW48, corresponds to Group I of the same classification. The high and low UBE2Q2-expressing cells also differ in other features such as amounts of production of growth factors and carcinoembrionic antigen (CEA).^24^ The production of CEA and some growth factors such as TGF-α, TGF-β, and platelet-derived growth factor (PDGF)-like material is significantly higher in SW742 cell line.^24^^,^^25^ However, further investigation needs to be conducted to find out whether UBE2Q2 expression can be considered a predictor of any of the factors that were used to group the cell lines.

Western blot analysis was employed to analyze the protein expression of UBE2Q2 in both cancerous and unaffected parts of the samples in our cohort of colorectal tissues. The data revealed overexpression of UBE2Q2 protein in the cancerous part of 65.11% of the tissue samples as compared to their unaffected parts. The results also showed no significant change between the cancerous and unaffected parts in 23.26% of the tumor specimens as well as the downregulation of the UBE2Q2 protein in the cancerous parts in 11.63% of the cases. This finding implies that the upregulation of UBE2Q2 may be a frequent and tumorigenic-related occurrence in CRC tissues. 

Our results demonstrated no significant association between immunoreactivity of UBE2Q2 and age, sex, degree of infiltration, or the tumor size of the cases in our cohort of CRC (table 1). Therefore, UBE2Q2 may be involved in the commencement but not the progression of CRC. The results of the previous studies have suggested that actin and other cytoskeleton proteins^15^ may be potential substrates for the UBE2Q2 protein. However, finding the correlation between the product(s) of UBE2Q2 gene and cancer development is a subject of further investigation. Possible tumorigenic-related roles that UBE2Q2 may play in the survival, shape, and migration of cells as well as their attachment to the substrate molecules are also potential outlooks for future studies.

Overexpression of UBE2Q2 in malignancies such as head and neck squamous cell carcinoma (HNSCC) and breast cancer as well as in most of the bone marrow samples from acute lymphoblastic leukemia patients has already been reported.^26^ Consistently, inactivation of UBE2Q2 is reported to cause cells to undergo prophase arrest and apoptosis in the M phase. Accordingly, it has been suggested that UBE2Q2 might act as an oncogene to promote the development of aneuploidy or malignancy in the M phase.^18^ The results of one study however, revealed that overexpression of UBE2Q2 negatively affects cell proliferation and anchorage-independent cell growth, which implies that UBE2Q2 may be a potential tumor suppressor.^27^ If confirmed, one possible explanation for these controversies is that the upregulation of UBE2Q2 in cancer tissues may be due to an inactive form and/or a dominant-negative isoform of the protein.

## Conclusion

Our data suggest that the novel human gene, UBE2Q2, may be a potentially useful tool in molecular diagnostic purposes and could be considered as a drug target for treating CRC in the future. However, the normal function of this gene and the role it may play in cancer require further investigation.
